# LIM kinases in cardiovascular health and disease

**DOI:** 10.3389/fphys.2024.1506356

**Published:** 2024-12-18

**Authors:** Olubodun M. Lateef, Christopher Foote, Gavin Power, Camila Manrique-Acevedo, Jaume Padilla, Luis A. Martinez-Lemus

**Affiliations:** ^1^ NextGen Precision Health, University of Missouri, Columbia, MO, United States; ^2^ Department of Medical Pharmacology and Physiology, University of Missouri Columbia, Columbia, MO, United States; ^3^ Department of Nutrition and Exercise Physiology, University of Missouri, Columbia, MO, United States; ^4^ Harry S. Truman Memorial Veterans’ Hospital, Columbia, MO, United States; ^5^ Department of Medicine, Division of Endocrinology, Diabetes and Metabolism, Columbia, MO, United States; ^6^ Center for Precision Medicine, Department of Medicine, University of Missouri, Columbia, MO, United States

**Keywords:** cardiovascular disease, vascular remodeling, arterial stiffening, atrial fibrillation, atherosclerosis, hypertension

## Abstract

The Lim Kinase (LIMK) family of serine/threonine kinases is comprised of LIMK1 and LIMK2, which are central regulators of cytoskeletal dynamics via their well-characterized roles in promoting actin polymerization and destabilizing the cellular microtubular network. The LIMKs have been demonstrated to modulate several fundamental physiological processes, including cell cycle progression, cell motility and migration, and cell differentiation. These processes play important roles in maintaining cardiovascular health. However, LIMK activity in healthy and pathological states of the cardiovascular system is poorly characterized. This review highlights the cellular and molecular mechanisms involved in LIMK activation and inactivation, examining its roles in the pathophysiology of vascular and cardiac diseases such as hypertension, aneurysm, atrial fibrillation, and valvular heart disease. It addresses the LIMKs’ involvement in processes that support cardiovascular health, including vasculogenesis, angiogenesis, and endothelial mechanotransduction. The review also features how LIMK activity participates in endothelial cell, vascular smooth muscle cell, and cardiomyocyte physiology and its implications in pathological states. A few recent preclinical studies demonstrate the therapeutic potential of LIMK inhibition. We conclude by proposing that future research should focus on the potential clinical relevance of LIMK inhibitors as therapeutic agents to reduce the burden of cardiovascular disease and improve patient outcomes.

## Graphical Abstract

**Figure:**
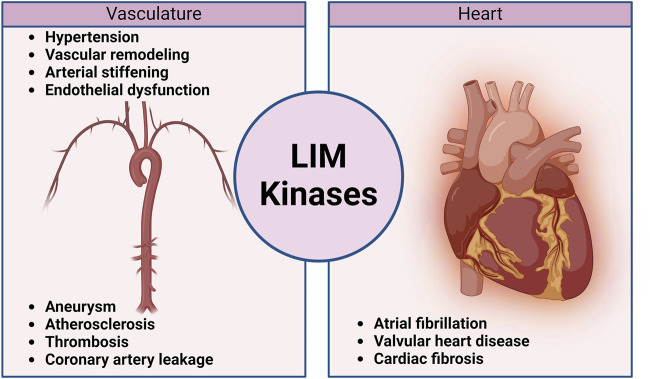
Schematic diagram illustrating the contribution of LIM kinases to the pathophysiology of cardiovascular diseases.

## 1 Introduction

The LIM (Lin-11, Islet-1, and Mec-3) kinase (LIMK) family of enzymes is considered a key regulator of cytoskeletal dynamics due to the ability of its members to promote actin polymerization. As multiple fundamental mechanisms of cellular function depend on proper actin cytoskeletal dynamics, it is crucial to understand the physiological, pathological, and potential therapeutic implications of LIMK activation and activity modulation. The LIMKs are canonical downstream substrates of the Rho-family small GTPase members, RhoA, Cdc42, and Rac1. In turn, the primary target of the LIMKs is the Actin Depolymerizing Factor (ADF)/Cofilin family of proteins, including cofilin 1, 2, and destrin, which will be referred to as cofilin in this review. The LIMKs can phosphorylate serine and tyrosine residues and have been demonstrated to phosphorylate cofilin at Ser3 as a primary target (Yang et al., 1998; Lagoutte et al., 2016; Salah et al., 2019). Ser3 phosphorylation of cofilin is an unusual but highly specific target of LIMK activity (Lagoutte et al., 2016; Salah et al., 2019) that inactivates cofilin and allows for filamentous actin (F-actin) to accumulate, as has been demonstrated with the use of LIMK overexpression or phospho Ser3 cofilin mimetics (Suurna et al., 2006; Zhao et al., 2008; Slee and Lowe-Krentz, 2013; Power et al., 2024b). In the cardiovascular system, F-actin is essential for maintaining the structural and functional integrity of vascular endothelial cells, smooth muscle cells, and cardiomyocytes. Therefore, disruptions in the mechanisms that control actin cytoskeletal dynamics can contribute to several cardiovascular diseases (Gorovoy et al., 2009; Yamin and Morgan, 2012; Ward and Crossman, 2014; Fediuk and Dakshinamurti, 2015; Pan et al., 2022). Additional evidence suggests that the LIMKs modulate the microtubular network via Rho-ROCK signaling pathways and the interaction between actin stress fibers with microtubules (Birukova et al., 2004b; Gorovoy et al., 2005). Because microtubules and F-actin exert opposing tensile forces to the cellular structural framework (vectors vs. tensors, respectively), the LIMKs can be considered central regulators of cytoskeletal tensegrity, i.e., the capacity of the cytoskeleton to stabilize the cell’s 3-dimensional structure by balancing forces of compression vs. tension, as poles, pegs and ropes do in a camping tent (Ingber, 2003b; Ingber, 2003a; Ingber et al., 2014; Gardiner, 2021). Overall, neuronal plasticity and cancer are the most extensive areas of study addressing the LIMKs’ role in cytoskeletal dynamics. Diverse roles for the LIMKs in the pathogenesis of neurological disorders (Cuberos et al., 2015), cancer manifestation (Yoshioka et al., 2003), and skin disorders (Honma et al., 2006) are reported in the literature. Comprehensive reviews in these areas are available, including those by Cuberos et al. (2015); Ben Zablah et al. (2021). However, reviews focused on the roles that the LIMKs play in the cardiovascular system and the potential applicability of LIMK inhibitors as therapeutic agents for cardiovascular disease are lacking. This is particularly important considering that cytoskeletal dynamics play significant roles in maintaining cardiovascular health. The cytoskeleton serves as the structural framework of all cardiovascular cells, maintaining cellular shape, providing mechanical integrity and resistance, and supporting the stability of intracellular proteins and attachments to the extracellular matrix (Gotlieb, 1990; Sequeira et al., 2014; Tang, 2018; Davis et al., 2023). In addition, cytoskeletal changes, particularly those involving F-actin and microtubules, have been linked to the development of specific vascular and cardiac disorders (Hein et al., 2000; Fediuk and Dakshinamurti, 2015; Subramanian et al., 2015; Morales-Quinones et al., 2020; Fang et al., 2022; Soares et al., 2022). Thus, this review is focused on the roles that the LIMKs play in cytoskeletal dynamics during physiological and pathological processes of cardiovascular cells and their potential therapeutic applications.

## 2 Regulation of the LIMKs

The LIMK family consists of two closely related members (LIMK1 and LIMK2), which are well-characterized substrates of the Rho-associated protein kinases (ROCK1 and 2). Both LIMKs have a unique organization of signaling domains, with two N-terminal LIM domains, an internal PDZ-like domain, and proline/serine-rich regions, followed by a C-terminal protein kinase domain. LIMK1 and LIMK2 have similar sequences with the tyrosine kinases despite being initially classified as serine/threonine kinases (Okano et al., 1995). Indeed, LIMK1 has been shown to phosphorylate serine and tyrosine residues (Yang et al., 1998; Ohashi et al., 2014; Lagoutte et al., 2016; Salah et al., 2019). Although the LIMKs are very similar, especially when comparing their kinase domains, there is mounting evidence that their regulatory pathways differ and contribute to unique and overlapping cellular and developmental activities (Scott and Olson, 2007). Like many other kinases, the LIMKs’ activity is enhanced by phosphorylation of their activation loop, and evidence suggests that the PDZ domain plays a role in auto-inhibiting the C-terminal kinase domain, as mutations in the PDZ domain increase LIMK catalytic activity (Casanova-Sepulveda et al., 2023).

### 2.1 LIMKs activation

**FIGURE 1:**
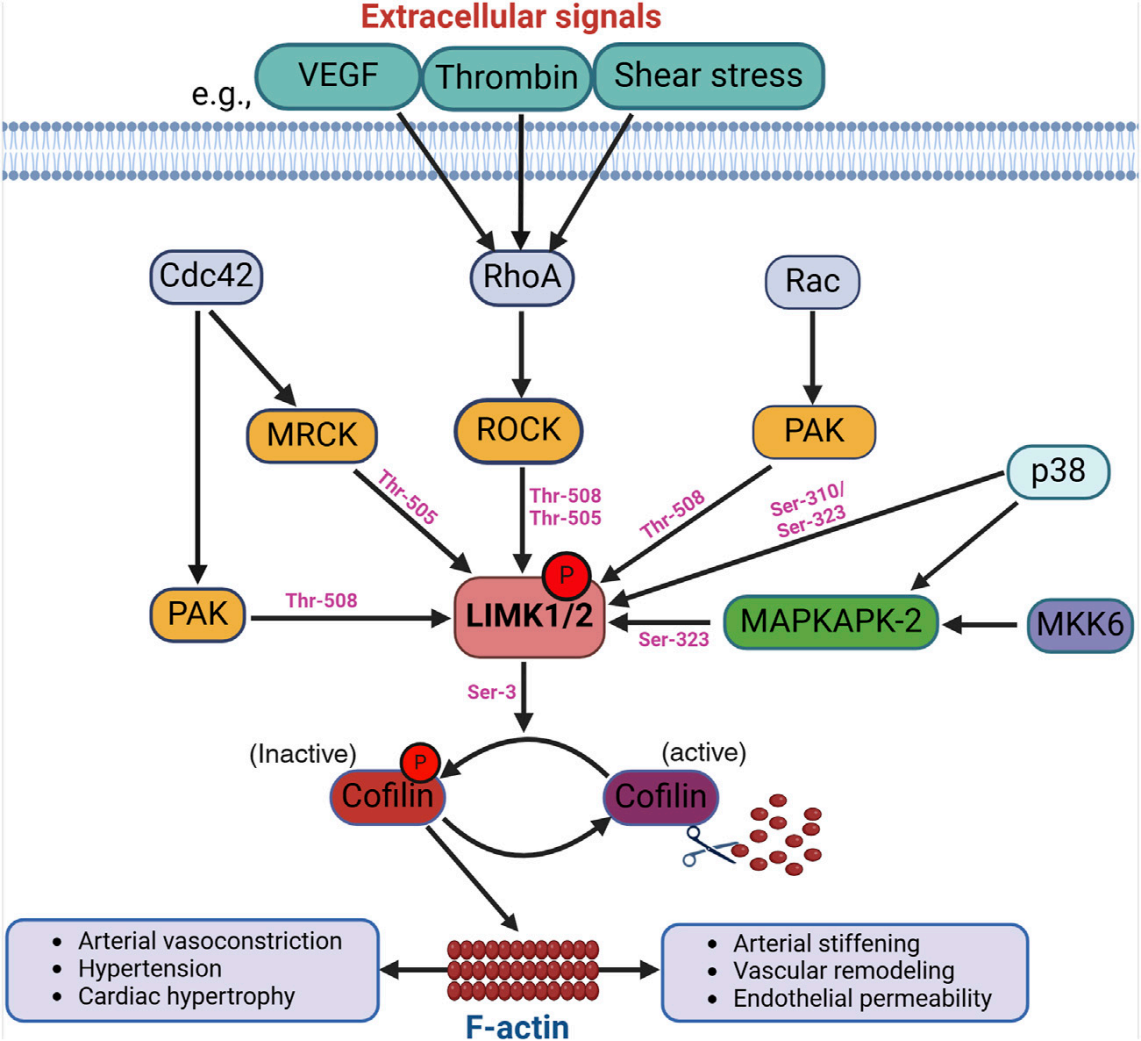
Activation of the LIMKs. Various upstream signaling pathways influence the LIMKs’ activation. For example, extracellular signals like VEGF, thrombin, and shear stress trigger the activation of RhoA, which subsequently phosphorylates ROCK. LIMK1 and LIMK2 undergo phosphorylation and activation by ROCK, and other kinases such as MRCK, PAK, p38, and MAPKAPK-2 at different phosphorylation sites as shown in the figure. LIMK activation leads to cofilin phosphorylation at Ser-3 resulting in the accumulation of F-actin. (VEGF: Vascular endothelial growth factor; ROCK: Rho-associated protein kinase; MRCK: myotonic dystrophy kinase-related Cdc42-binding kinases; Cdc42: cell division control protein 42; PAK: p21-activated kinases; MAPKAPK-2: mitogen-activated protein kinase-activated protein kinase-2).

The LIMKs are predominantly activated by diverse kinases positioned downstream from members of the Rho family of small GTPases (Villalonga et al., 2023). The best-characterized activation process of the LIMKs is contingent upon RhoA GTPase activation of ROCK with the subsequent phosphorylation of LIMK1 and LIMK2 at Thr-508 and Thr-505, respectively (Ohashi et al., 2000; Amano et al., 2001). Activation of the LIMKs downstream of Cdc42 and Rac1 also occurs via intermediary kinases. Current data suggest that LIMKs’ activation downstream of Cdc42 involves the myotonic dystrophy kinase-related Cdc42-binding kinases (MRCK), which phosphorylate LIMK2 at Thr-505 (Sumi et al., 2001). For both Cdc42 and Rac1, the effectors include the p21-activated kinases (PAK), which phosphorylate LIMK1 at Thr-508 (Edwards et al., 1999). Indeed, PAKs increase their activity when associated with GTP-bound Cdc42 or Rac1 through their Cdc42 and Rac1-binding domain, and PAKs’ activity has been shown to phosphorylate and activate the LIMKs (Figure 1).

Evidence for additional pathways by which the LIMKs become activated includes data indicating that p38 mitogen-activated protein kinase (MAPK) regulates LIMKs’ phosphorylation status via MAPK-activated protein kinase-2 (MAPKAPK-2) (Scott and Olson, 2007). In endothelial cells, such activation of LIMK1 does not require Thr-508 phosphorylation, as it has been shown that vascular endothelial growth factor (VEGF) stimulation leads to MAPKAPK-2-dependent phosphorylation of LIMK1 at Ser-323 between the PDZ and kinase domains (Kobayashi et al., 2006). Further evidence indicates that the LIMKs are also directly phosphorylated by p38 at Ser-310 and Ser-323; however, the effect of Ser-310 phosphorylation on LIMKs’ activity appears negligible. This further suggests that kinases such as p38 activate the LIMKs indirectly via MAPKAPK-2 activity. Several additional kinases activate the LIMKs (Manetti, 2012; Villalonga et al., 2023), some with an apparent specificity for one of the two different isoforms. These kinases include protein kinase A (PKA), MKK6, and Aurora kinase A (AURKA). The LIMKs also appear to dimerize and undergo autophosphorylation or transphosphorylation processes induced by heat shock protein (HSP)-90 and tropomyosin-related kinase B (TrkB) (Li et al., 2006; Dong et al., 2012). These processes, in turn, favor the translocation of the LIMKs from the cytosol to the cell membrane. Additional ways for the LIMKs to augment their capacity to phosphorylate cofilin include their increased expression resulting from p53 activity and the interaction of LIMK1 with the cyclin-dependent kinase inhibitor p57^krp2^ (Hsu et al., 2010). It remains to be fully elucidated which activation pathways are specific for the different LIMK isoforms and the role of such pathways in cell-specific phenomena. In-depth recent reviews have been published on the diverse mechanisms that activate the LIMKs, how different mechanisms interact to increase or decrease the capacity of the LIMKs to phosphorylate cofilin, and which mechanisms require further corroboration or represent fruitful avenues for research endeavors (Chatterjee et al., 2022; Jiang et al., 2023; Villalonga et al., 2023; Casanova-Sepulveda and Boggon, 2024). Currently and consistent with their role in modulating cytoskeletal dynamics, the kinases downstream of Rho, Cdc42, and Rac1 are considered the primary activators of the LIMKs.

### 2.2 LIMKs inactivation

Typically, proteins subject to phosphorylation are dephosphorylated by phosphatases that return them to their unphosphorylated state. Slingshot 1 (SSH1), previously identified as a cofilin phosphatase, is among the phosphatases responsible for dephosphorylating and deactivating LIMK1 (Soosairajah et al., 2005; Gomez-Moron et al., 2024). In this process, SSH1 directly interacts with the kinase domain of LIMK1, facilitating the dephosphorylation of Thr-508 and consequently reducing the downstream phosphorylation of cofilin by LIMK1. Notably, SSH1 demonstrates a higher affinity for LIMK1 than LIMK2, suggesting that the phosphatase provides a differential regulation of these two kinases (Scott and Olson, 2007). Data also indicate that kinases that phosphorylate and activate the LIMKs further enhance LIMKs’ activities by dampening SSH1’s dephosphorylation capacity (Scott and Olson, 2007). For example, PAK4, which phosphorylates the LIMKs, also phosphorylates and inactivates SSH1 (Soosairajah et al., 2005). This indicates that the activity of the LIMKs is tuned by processes that finely control the phosphorylation status of the enzymes (Scott and Olson, 2007). These processes also include the status of the actin cytoskeleton, as low levels of F-actin in the cell can decrease SSH1 activity and thus promote the activation of LIMK1 and the polymerization of F-actin (Kurita et al., 2008).

**FIGURE 2:**
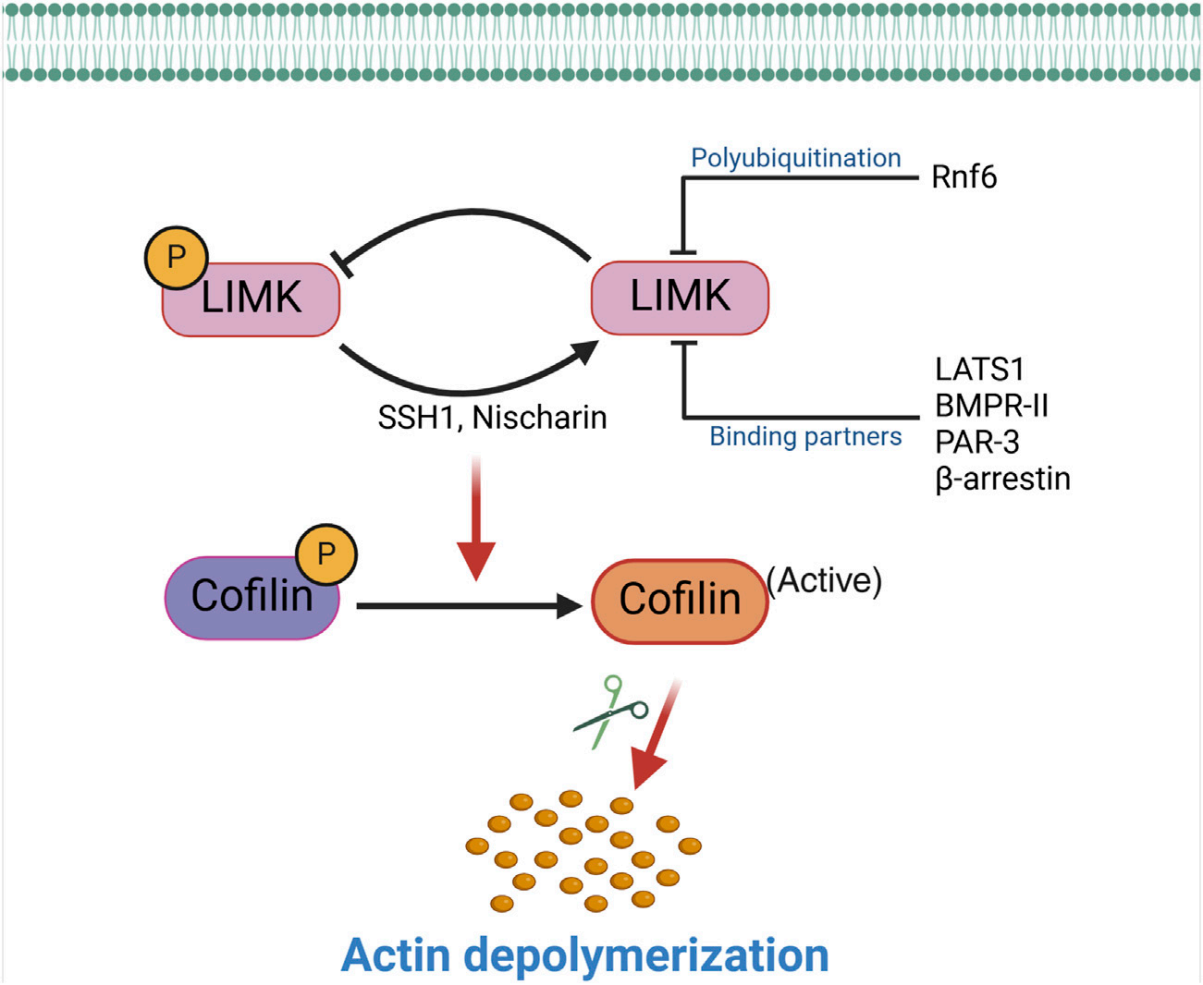
Inactivation of the LIMKs. The activity of the LIMKs is inhibited through dephosphorylation by SSH1, Nischarin. LIMKs are polyubiquitinated and degraded by Rnf6, while interactions with binding partners (LATS1, BMPR-II, PAR-3, and β-arrestin) inhibit their phosphorylation and prevent them from phosphorylating cofilin. This negative regulation of LIMK reduces cofilin phosphorylation, thereby enhancing the actin-severing activity of cofilin. (SSH1: Slingshot 1; LATS1: Large tumor suppressor kinase 1; PAR-3: Protease-activated receptor-3; Rnf6: Ring finger protein 6; BMPR-II: Bone morphogenetic protein receptor II).

Other mechanisms, in addition to the phosphatase activity of SSH1, reduce the capacity of the LIMKs to phosphorylate cofilin. For example, bone morphogenetic protein (BMP) and its type II receptor downregulate the activity of LIMK1 via the interaction of the enzyme with the intracellular tail of BMPR-II. This inhibits LIMK1’s capacity to phosphorylate cofilin, which can be reversed by adding a BMP ligand to disrupt the interaction between LIMK1 and BMPR-II, leading to the subsequent activation of the enzyme (Foletta et al., 2003). Other molecules also participate in inactivating the LIMKs either by sequestering the LIMKs, thereby preventing phosphorylation at Thr-508 or Thr-505, or by facilitating the dephosphorylation of those activation sites (Figure 2). For example, Nischarin, a scaffolding protein that interacts with integrin α5β1 and participates in intracellular signaling (Alahari et al., 2000), also interacts and forms a complex with LIMK1 (Ding et al., 2008). This interaction leads to LIMK1 dephosphorylation and inactivation (Ding et al., 2008). Large tumor suppressor kinase 1 (LATS1) also interacts with LIMK1, rendering the enzyme inactive and reducing the phosphorylation level of downstream cofilin. Other cellular components that interact with the LIMKs and reduce their lifespan or kinase capacity include β-arrestin, the RING finger E3 ubiquitin ligase Rnf6, and PAR-3 (a protein involved in forming tight junctions) (Tursun et al., 2005; Chen and Macara, 2006; Zoudilova et al., 2007). Ultimately, processes that impede the phosphorylation of the LIMKs increase the capacity of phosphatases to dephosphorylate the enzymes, reduce their stability and half-life, or dephosphorylate cofilin to promote the severance of F-actin commonly associated with LIMKs’ inhibition. Phosphatases that directly dephosphorylate cofilin include SSH1, protein phosphatase-1 and -2A (PP1, PP2A), and chronophin (Villalonga et al., 2023).

## 3 LIMKs’ roles in the vasculature

The actin cytoskeleton is constantly remodeling via actin polymerization and depolymerization processes that fortify or weaken (degrade) F-actin stress fibers. Although this is particularly evident in motile cells and cells that constantly protrude and retract cellular extensions, vascular differentiated and de-differentiated cells also undergo constant changes in actin polymerization and depolymerization. Consequently, studies on the roles of LIMKs in the vasculature have focused on the effects of actin cytoskeletal dynamics in vascular remodeling and function, including but not limited to arterial stiffening, vasoconstriction, and vascular permeability. Most mechanistic studies in these research areas have been performed using LIMK inhibitors and not via knock down, knock out, or overexpression of the LIMKs’ gene products. The cardiovascular pathology present in Williams–Beuren syndrome (a genetic disorder caused by heterozygous deletion of genes including *LIMK1* at chromosome 7q11.23) appears to depend on the heterozygous deletion of the elastin gene and not that of LIMK1. In addition, published data on knock-out models do not indicate that deletion of LIMK1 or LIMK2 produces an overt vascular phenotype. However, to the best of our knowledge, no specific analyses of vascular structure and function under basal or stressed conditions have been reported using such genetic models. It is also unclear which isoforms of the LIMKs or cofilin predominate in each vascular cell type. Although data using LIMK inhibitors indicate that LIMK activity modulation affects vascular function and structure, most of the inhibitors used do not distinguish family members, limiting the attribution of the phenomena studied to a specific LIMK isoform.

### 3.1 LIMKs and arterial remodeling

Arterial remodeling plays a critical role in cardiovascular physiological adaptations and the development and progression of cardiovascular diseases. Remodeling constitutes an overall important adaptive feature for maintaining blood vessel integrity. In broad terms, arterial remodeling refers to any change in vessel wall structure. However, it has been further defined as a change in the internal passive diameter of a vessel while under a specific intravascular pressure (Mulvany et al., 1996). Thus, a smaller passive internal diameter in a vessel represents inward remodeling, while a larger diameter represents outward remodeling. This is further characterized by the wall’s cross-sectional area (CSA), such that a reduction in CSA is classified as hypotrophic, no change as eutrophic, and an increase as hypertrophic remodeling. This definition of vascular remodeling was first put forth by Mulvany et al. (1996) and has been mostly used for blood vessels in the microcirculation. In larger arteries where the presence of atherosclerotic plaques or neointimal formation may confound the internal diameter of an artery, the terms outward or inward remodeling are more commonly used to describe changes in the CSA contained within the external elastic lamina (Ward et al., 2000). Arterial remodeling is initiated by complex pathophysiological mechanisms that directly impact the vascular wall’s cellular and non-cellular (extracellular matrix, ECM) components. These mechanisms include endothelial cell dysfunction, elastin and collagen content changes, vascular smooth muscle cell (VSMC) structure and function impairment, fibrosis, and calcification (Van Varik et al., 2012). Furthermore, multiple publications indicate that arterial remodeling precedes and participates in the development and progression of cardiovascular conditions such as hypertension (Weisbrod et al., 2013), aneurysm (Perissiou et al., 2019), or atherosclerosis (Palombo and Kozakova, 2016) and that changes in actin polymerization within VSMCs play an essential part in the early phases of arterial remodeling as well as in the maintenance of the remodeled state. In this regard, LIMK activity has been studied in vascular remodeling, as it highlights its capacity to enhance the formation and stabilization of F-actin by phosphorylating cofilin and suppressing its F-actin severing activity (Foote et al., 2016; Morales-Quinones et al., 2020).

Inward remodeling of the arterial vasculature is commonly associated with arterial stiffening. The stiffening of the vasculature has been widely attributed to elastin fragmentation, excessive collagen deposition, and crosslinking (Zhang et al., 2016; Hayashi and Hirayama, 2017; Aroor et al., 2019). However, an increasing body of evidence indicates that the mechanical properties of VSMCs also contribute to arterial stiffness (Sehgel et al., 2015; Martinez-Lemus et al., 2017). For example, Staiculescu et al. (2013) demonstrated that inhibition of VSMC actin polymerization prevents the development of inward remodeling in resistance arteries and further showed that F-actin depolymerization increased arterial passive diameter in inwardly remodeled vessels. This indicates that actin polymerization is a critical player in the mechanical processes that increase arterial stiffness and inward vascular remodeling. The polymerization and depolymerization of actin are tightly controlled processes, and many signaling factors, including kinase/phosphatase activity, temperature, Ca^2+^, and pH, affect the function of different actin-binding proteins. All eukaryotic cells express the actin depolymerization cofilin family of proteins needed for actin filament turnover. These proteins weakly sever actin filaments without capping their ends, thereby increasing the number of uncapped filament ends available for polymerization and depolymerization (Goyal et al., 2013). As cofilin’s primary activity is to sever F-actin cytoskeletal stress fibers, inactivation of LIMK activity as the upstream regulator of cofilin phosphorylation may offer potential therapeutic strategies for preventing vascular inward remodeling and arterial stiffening. Indeed, recent research by Morales-Quinones et al. shows that LIMK inhibition reduces vasoconstriction-induced arterial stiffening by lowering the amount of F-actin and the stiffness of VSMCs (Morales-Quinones et al., 2020). Moreover, inhibition of LIMK prevented arterial stiffening and inward remodeling in isolated human visceral arteries exposed to prolonged vasoconstriction (Morales-Quinones et al., 2020). In that study, fluorescence images of omental arteries from hypertensive subjects showed increased F-actin stress fiber content and cofilin phosphorylation compared to non-hypertensive patients. LIMK inhibition also prevented the increased cortical stiffness observed after prolonged exposure to vasoconstrictor agonists in cultured VSMCs. Using a mouse model of hypertension, contralateral ears treated with a LIMK inhibitor prevented the reduction in vascular diameter following prolonged angiotensin II infusion (Morales-Quinones et al., 2020). In addition, recent findings by Power et al. (2024b) indicate that LIMK inhibition reduces endothelial cell stiffness by decreasing phosphorylated cofilin levels and consequently reducing F-actin stress fibers. These data suggest that targeting LIMK may lead to novel strategies to modulate arterial stiffness and ameliorate vascular remodeling in hypertension.

Arterial stiffening is correlated with end-organ damage, and several cardiovascular diseases are associated with increased vascular stiffness due to consequent increases in impedance and impaired vascular elasticity (Cheung, 2010). Changes in the characteristics of major vessel wall components, such as elastin, VSMCs, and collagen, cause aging-associated arterial stiffening. However, cumulative data on aging and obesity indicate that VSMC stiffness is a major driver of arterial stiffening (Qiu et al., 2010; Zhu et al., 2012; Ramirez-Perez et al., 2022). Therefore, considering that VSMC stiffness is modulated by cofilin phosphorylation through the RhoA-ROCK-LIMK signaling axis (Bravo-Cordero et al., 2013; Swiatlowska et al., 2022), modulation of LIMK activity may represent a novel therapeutic target to reduce arterial stiffness associated with aging and obesity.

### 3.2 LIMKs and arterial aneurysms

Aortic or intracranial aneurysms are defined as structural dilations that occur at weak points along the aorta or the cerebral vasculature, respectively. Four events are considered leading to aneurysm formation, including lymphocyte and macrophage infiltration into the vessel wall, vascular wall elastin and collagen destruction by proteases, VSMC loss within the media, and neovascularization (Ailawadi et al., 2003). Notably, rupture of aortic aneurysms has been associated with a mortality ranging from 50% to 80%, while subarachnoid hemorrhage is the most common consequence of intracranial aneurysm rupture, with a mortality rate of 27%–44% (Zhang et al., 2003; Kuivaniemi et al., 2015). Given the importance of the structural integrity of the vascular wall in precipitating aneurysm, the role of the cytoskeleton and its regulators has been investigated. LIMK has been demonstrated to promote aneurysmal formation. Single nucleotide polymorphisms in LIMK1 at chromosome 7q11 may cause intracranial aneurysm formation by affecting the structural support of the vascular wall (Akagawa et al., 2006). More specifically, the single nucleotide polymorphism rs6460071 in LIMK1 was associated with an increased risk of intracranial aneurysm formation (Low et al., 2011). This mutation reduces the promoter activity of *LIMK1* and a consequent decrease in LIMK1 protein levels, leading to the malformation of cerebral blood vessels and an increased incidence of intracranial aneurysms (Akagawa et al., 2006). However, it remains to be determined whether a reduction in LIMK activity alone can promote aneurysm formation. In a mouse model of ascending aortic aneurysms, both SSH1 abundance and cofilin dephosphorylation were increased during the phase of aneurysm initiation. This coincided with a reduction in F-actin and increased G-actin content within the developing aneurysm in the ascending aorta (Yamashiro et al., 2015). Paradoxically, there were also increases in RhoA abundance and activity and LIMK phosphorylation in the ascending aortas, ascribed to increased angiotensin II signaling in the mouse model of aneurysm used. The activity of LIMK is not well characterized in other models of aneurysm formation. However, reductions in F-actin and increases in G-actin are plausible mechanistic hypotheses for the weakening process that occurs in the aortic wall during the initiation stage of aneurysm development, which would likely implicate changes in the modulation of cofilin by the LIMKs.

In support of a prominent role for enzymes that affect both the cytoskeleton and ECM in aneurysm pathophysiology, tissue-type transglutaminase (TG2) expression was shown to be increased in the maximally dilated portion of human abdominal aorta aneurysmal samples compared to nondilated segments of the aorta (Shin et al., 2013). TG2 has been shown to inhibit MMP-2, -9, and tumor necrosis factor (TNF)-α in primary cultures of human abdominal aortic aneurysm-derived smooth muscle cells, supporting the notion that TG2 stabilizes the ECM and prevents the progression of abdominal aortic aneurysm (Shin et al., 2013). There is further evidence that members of a TG2-Rho-ROCK-LIMK-cofilin pathway may be involved in arterial aneurysm development, but how TG2 directly regulates LIMK activity in aneurysm development and progression remains poorly understood. In a mouse model of abdominal aortic aneurysm induced by intraluminal elastase and extraluminal calcium chloride exposure *in vivo*, TG2, TNF-α, MMP-2, and MMP-9 mRNA expression were increased in the acute phase compared to the chronic phase of the disease (Munezane et al., 2010). The mRNA expression of key factors promoting aneurysm formation, such as TNF-α, MMP-2, and MMP-9 in cultured aneurysmal tissue was decreased by exogenous TG2 exposure and increased by cystamine, a competitive inhibitor of TG2. This suggests a role for TG2 activity in ECM protection during the chronic phase of aneurysmal progression (Munezane et al., 2010). Because TG2 activity has also been shown to participate in VSMC actin dynamics and modulation of LIMK activity (Munezane et al., 2010), TG2 activation likely provides additional protection against aneurysmal development and progression via VSMC cytoskeletal modulation. In this scenario, inhibition or decreased expression of TG2 promotes the weakening of the vessel wall, leading to aneurysmal formation, while activation of TG2 protects against the development, progression, and potential rupture of the aneurysm. A few studies have examined the role of TG2 in abdominal aortic aneurysms (Munezane et al., 2010; Shin et al., 2013; Griffin et al., 2021), but there is a lack of information on how LIMK directly regulates the aneurysmal process. Further studies should determine the molecular mechanisms by which a TG2-dependent LIMK activity pathway participates in aneurysm pathophysiology.

### 3.3 LIMKs and atherosclerosis

The dynamic remodeling of the actin cytoskeleton via LIMK activity is crucial for cell migration, gene expression, and morphogenesis (Scott and Olson, 2007; Manetti, 2012; Mizuno, 2013; Ohashi et al., 2014). Cell migration is essential for maintaining and establishing cellular organization, wound repair, tissue homeostasis, and a proper immune response (Trepat et al., 2012). Nonspecific electrostatic interactions and specific binding molecules such as cadherins, selectins, and integrins mediate cell adhesion to the ECM or nearby cells (Trepat et al., 2012). Cytoskeletal proteins such as α-actinin (Choi et al., 2008), filamin (Nagano et al., 2002), talin (Burridge and Connell, 1983), and tensin (Lo et al., 1994) link integrins to the actin cytoskeleton. Accordingly, the actin cytoskeleton is remodeled during VSMC migration in response to signals from cell surface receptors such as the integrins. This reorganization of the ECM-integrin-cytoskeleton axis causes the leading edge of VSMCs to protrude either along a path of variable adherence to the ECM or in the direction of a chemotactic signal (Gerthoffer, 2007). In addition to remodeling the cytoskeleton and focal adhesion disintegration at the trailing edge, contraction caused by actomyosin molecules in the cytoplasm propels the cell forward. By severing F-actin and expanding the number of F-actin portions available for actin polymerization, LIMK activity plays a significant role in cell proliferation and migration.

**FIGURE 3:**
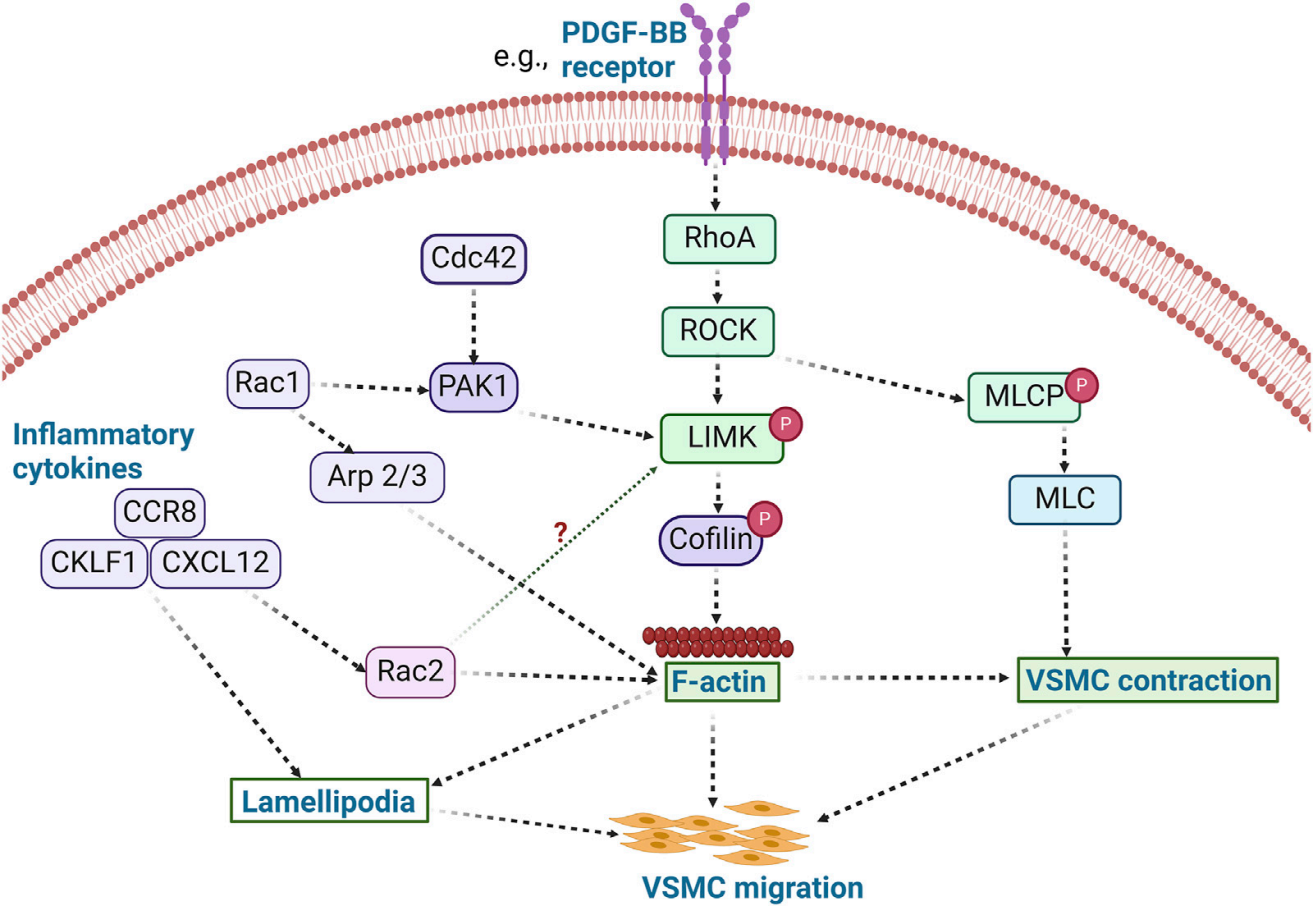
LIMK activity promotes VSMC migration. The activation of RhoA/ROCK by stimuli such as PDFG-BB leads to the phosphorylation of LIMK and inactivation of cofilin, thereby promoting actin polymerization. Concurrently, ROCK phosphorylates MLCP and inhibits its activity, resulting in increased phosphorylation of MLC and subsequent VSMC contraction. Activation of inflammatory cytokines such as CCR8, CKLF1, and CXCL12 also modulates cell migration via lamellipodia formation. These inflammatory processes likely promote VSMC migration in a LIMK-dependent manner. Whether inflammatory cytokines activate LIMK via Rac2 activation remains unknown. (PDFG-BB: Platelet derived growth factor BB; Arp 2/3: Actin related protein 2/3; CCR8: chemokine receptor-8; CKLF1: Chemokine-like factor 1; CXCL12: C-X-C motif chemokine 12; MLCP: Myosin light chain phosphatase).

Indeed, modulation of LIMK activity and its associated pathways affects cell migration, including that of VSMCs. For example, use of the non-selective inhibitor of LIMK activity, damnacanthal, reduced breast carcinoma cells migration and invasion (Ohashi et al., 2014). Treatment with damnacanthal also prevented CXCL12-induced lamellipodium formation and suppressed migration in Jurkat cells through inhibition of LIMK1. A similar phenotype was reported by Nishita et al., who showed that LIMK1 knockdown suppressed chemokine-induced lamellipodium formation and cell migration (Nishita et al., 2005). Chemokine-like factor 1 (CKLF1) and chemokine receptor-8 (CCR8) have been shown to play an essential role in the migration and proliferation of VSMCs in vascular inflammation (Haque et al., 2004; Zhang et al., 2013) (Figure 3), and inhibition of CKLF1 can be used as a therapeutic target for the prevention of atherosclerosis. Furthermore, RhoA activates ROCK1 and ROCK2 in VSMCs, and non-selective inhibition of ROCK by fasudil or C3 exoenzyme impairs VSMC migration (Negoro et al., 1999; Seasholtz et al., 1999; Liu et al., 2002). The myosin binding component of myosin light chain phosphatase is phosphorylated by ROCK, which lowers phosphatase activity. ROCK can also directly phosphorylate Ser19 on myosin light chains *in vitro* (Amano et al., 1996), and in addition to its effects on myosin II phosphorylation (Gerthoffer, 2007), ROCK enhances actin polymerization by activating LIMK. Thus, various VSMC chemotactic signals that induce RhoA/ROCK activation, cytoskeletal remodeling, and VSMC migration (Raines, 2004) are potentially present in atherosclerosis and neointimal formation and represent processes likely modulated by LIMK activity.

The primary physiological functions of VSMC migration are vascular development and the maintenance of vascular integrity (Gerthoffer, 2007). In contrast, the pathophysiological migration and proliferation of VSMCs occur in response to vascular injury and atherogenesis (Gerthoffer, 2007). In this context, the differentiation and migration of VSMCs contribute to the pathological thickening of the arterial intima. Indeed, at the root of atherosclerosis and restenosis is the migration of VSMCs from the medial to the intimal layer of vessels (Chava et al., 2009; Gomez and Owens, 2012). Evidence indicates dynamic variations in LIMK expression and cofilin activity in VSMCs exist (Dai et al., 2006). However, new research on the role of LIMK activity in regulating VSMC migration in atherosclerotic plaque development and neointimal formation is warranted. In addition, vascular tone may be modulated by the differentiation of VSMCs from their contractile to a secretory or osteogenic phenotype. This promotes vascular wall calcification and secretion of proinflammatory cytokines such as platelet-derived growth factor (PDGF) (Winkles and Gay, 1991; Chang et al., 2014), critical characteristics of atherosclerosis development, progression, and complication.

PDGF plays a crucial role in atherosclerosis, vascular healing, and restenosis by promoting VSMC migration. While the stimulation of VSMC migration by PDGF is accompanied by cofilin activation, it has been demonstrated that dual regulation of cofilin activity by LIMK and SSH1 controls PDGF-induced migration of VSMCs (San Martin et al., 2008). PDGF activates both LIMK and the novel phosphatase SSH1L, but the activity of SSH1L supersedes LIMK activity, leading to the dephosphorylation of cofilin and VSMC migration. Indeed, inhibition of cofilin dephosphorylation by siSSH1L prevents cell migration, suggesting that the dual regulation of cofilin activity represents a finely tuned control of directional migration in which SSH1L plays a significant role in the maintenance of cofilin activity during PDGF-induced VSMC migration (San Martin et al., 2008). Similarly, in CXCL12-induced cell migration, LIMK1 and SSH1L are both activated in the process and mediate spatiotemporal regulation of cofilin. Ablation of either LIMK1 or SSH1L impairs chemokine-mediated cell migration (Nishita et al., 2005). LIMK1 is implicated in lamellipodium formation early in the migratory process. In contrast, the subsequent activation of SSH1L is posited to serve a dual purpose in restricting lamellipodia extension to one direction by retarding the growth of additional lamellipodia at other areas of the cell and then becoming discretely active at the leading edge of the lamellipodia to facilitate cyclic retraction and extension of the remaining lamellipodia. This proposed mechanism of cell migration induced by chemokines is more nuanced than that of several other studies that demonstrate cofilin phosphorylation promotes F-actin formation and cell migration.

VSMC migration induced by PDGF has also been associated with the activity of Rac-dependent induction of peripheral actin accumulation and membrane ruffling (Doanes et al., 1998). LIMK1 is involved in the lamellipodia formation induced by Rac1 (Sumi et al., 1999), while inflammation-activated Rac2 cooperates with the cytokine-inducible scaffold protein and allograft inflammatory factor to facilitate VSMC migration (Tian and Autieri, 2007). Similarly, PAK activity contributes to VSMC migration through Rac/Cdc42 GTPase signaling (Edwards et al., 1999). PAK phosphorylates LIMK and promotes actin polymerization when LIMK phosphorylates cofilin. In contrast, myosin phosphorylation is prevented when PAK phosphorylates myosin light-chain kinase (Edwards et al., 1999). Although it has been shown that VSMC migration is significantly inhibited by the expression of kinase-inactive PAK (Dechert et al., 2001), the predominant positive or negative effects of PAK and downstream LIMK activity on cytoskeletal filaments, myosin, and focal adhesions during VSMC migration remain poorly understood. All these indicate that Rac/Cdc42 and RhoA signaling are active participants in VSMC migration. Further studies should be conducted to accurately decipher the spatiotemporal activation of the LIMKs during the regulation of VSMC migration in response to diverse stimuli associated with atherosclerosis and neointima formation.

### 3.4 LIMKs and endothelial cell mechanotransduction

The survival and functional maintenance of living cells are linked to the cells’ ability to perceive and respond to mechanical forces and the characteristics of their immediate extracellular microenvironment (Ingber, 2006; Janmey and McCulloch, 2007). The vascular endothelium plays critical homeostatic roles such as modulating macromolecular permeability, inflammatory responses, regulation of vascular remodeling, thrombosis, and vascular tone regulation in response to mechanical and chemical stimuli (Chiu and Chien, 2011; Davis et al., 2023). The two main mechanical forces acting upon the endothelium are the stretch caused by blood vessel deformation and the shear stress brought about by blood flow (Ali and Schumacker, 2002; Davis et al., 2023; Power et al., 2024a). These mechanical forces and associated mechanotransduction responses maintain endothelial cell morphology, signaling, and function under physiological conditions. However, during pathological conditions, altered mechanical forces and cellular responses participate in remodeling the structure of the ECM and the endothelium (Ingber, 2002). As a result, endothelial cells change their expression of molecules, such as the integrin receptors at cell-cell junctions and cell-matrix adhesions, to modulate the detection of mechanical stimuli, convert them into electrical or biochemical signals, and impact endothelial cell function (Dorland and Huveneers, 2017; Seetharaman and Etienne-Manneville, 2018). During this process, the RhoA/ROCK/LIMK pathway transduces mechanical signals from integrins to the cytoskeleton. Shear stress-induced ROCK/LIMK activity also facilitates endothelial cell migration by enhancing the traction force between the endothelial plasmalemma and the underlying substrate (Liu et al., 2018). Indeed, a growing body of evidence shows that the relationship between integrins, Rho GTPases, and the actin cytoskeleton is crucial for the mechanotransduction of shear stress (Shyy and Chien, 2002) and that endothelial shear stress is essential for maintaining vascular wall homeostasis (Davies, 1995; Power et al., 2024a).

Shear stress induces Rho activation via multiple pathways, one of which includes integrins bound to fibronectin (Lin et al., 2003). It also causes a rapid increase in the kinase activity of ROCK and a sustained activation of LIMK2 (Lin et al., 2003). In cultured endothelial cells, integrin activation by shear engages the Rho-ROCK-LIMK-cofilin pathway to regulate the activity of sterol regulatory element binding proteins (SREBPs), which are important transcription factors responsible for the regulation of fatty acid, cholesterol, and triglyceride synthesis (Lin et al., 2003). Lin et al. showed that ROCK, LIMK, and cofilin are important mediators of shear stress-induced Rho activation of SREBP2 and that dominant negative Rho, RhoN19, prevents shear stress-induced SREBP2 nuclear translocation (Lin et al., 2003). The activation of SREBPs by shear stress may represent a novel mechanism by which mechanotransduction can be used as a tool to regulate SREBPs activity and the synthesis of lipids and sterols *in vivo* (Lin et al., 2003). This may be achieved via modulation of the Rho-ROCK-LIMK signaling cascade, as shear stress activates SREBPs via Rho by triggering the upstream mechanosensing process that involves integrins and ECM proteins. Indeed, Rho activity must be adequately regulated for shear stress to promote cell alignment and the creation of stress fibers, as either inhibition of Rho or activation of its mutant (RhoV14) causes a reduction in cell alignment (Li et al., 1999; Tzima et al., 2001). Thus, it is plausible that endothelial remodeling and lipid and sterol homeostasis may be regulated by tuning the Rho-ROCK-LIMK-cofilin pathway.

### 3.5 LIMKs in endothelial cell migration, vasculogenesis, and angiogenesis

Endothelial cell migration is essential for both angiogenesis and vasculogenesis and for restoring vessel integrity after damage (Michaelis, 2014). The migration of endothelial cells is regulated by mechanotactic, haptotactic, and chemotactic stimuli that modulate the progression and directionality of the migrating cells. This motile process requires the activation of various signaling cascades that converge on cytoskeletal remodeling (Lamalice et al., 2007). Thus, actin remodeling and focal adhesion dynamics are critical components of endothelial cell migration. Indeed, cell migration depends on the constant remodeling of the actin cytoskeleton into lamellipodia, stress fibers, and filopodia. Accordingly, endothelial cell migration is regulated by ROCK and requires the contraction of actomyosin, the rearrangement of stress fibers, and the dynamic formation and disassembly of focal adhesions (Lin et al., 2003; Wehrle-Haller, 2012).

**FIGURE 4:**
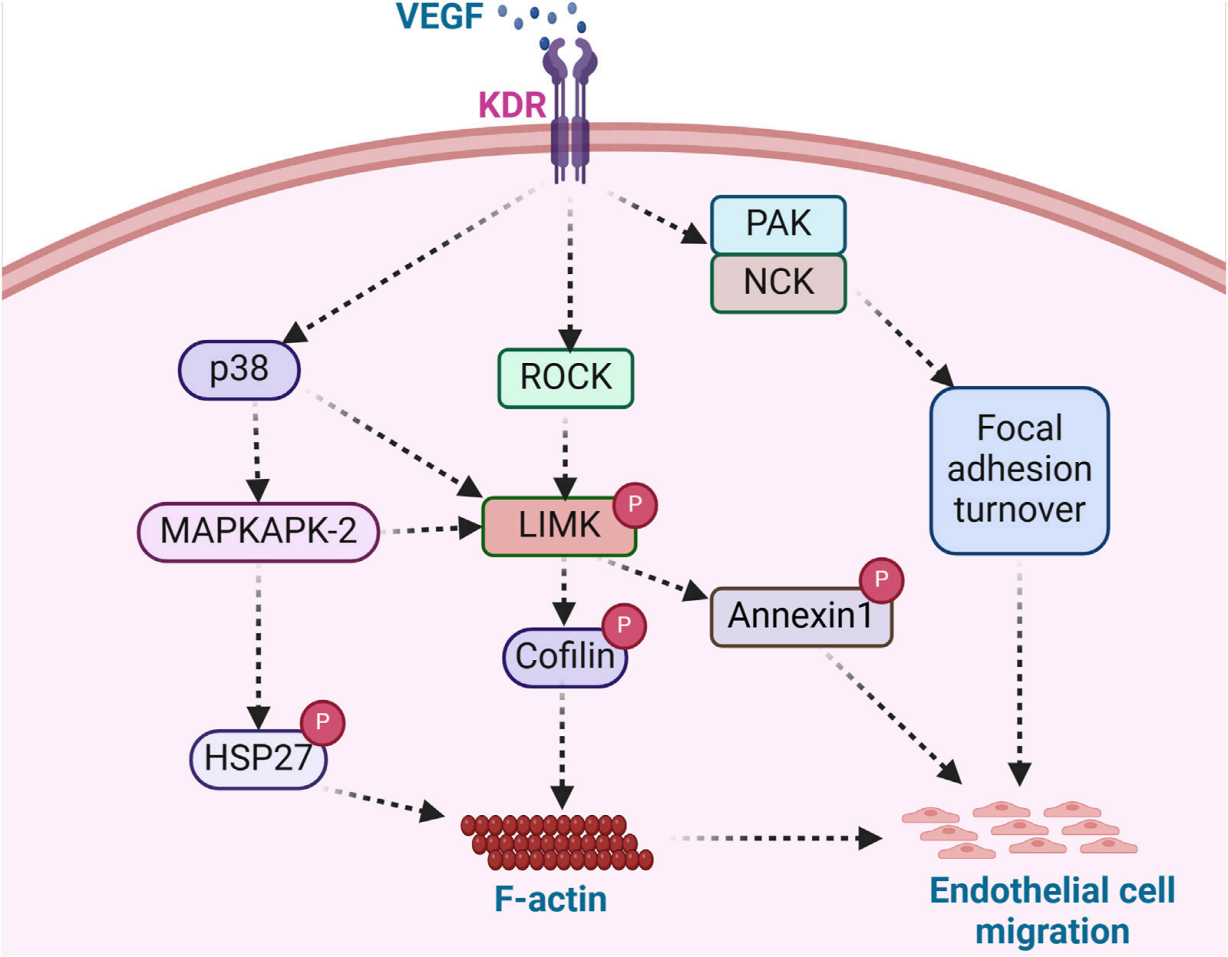
LIMK acts as an integral regulator of endothelial cell migration. Angiogenesis modulators such as VEGF bind surface receptors in endothelial cells and trigger the activation of ROCK, PAK/NCK, and p38 signaling pathways. ROCK activation, stimulated by VEGF, facilitates LIMK-induced cofilin deactivation and subsequent actin polymerization, while PAK/NCK activation reinforces focal adhesion turnover. LIMK-mediated annexin1 phosphorylation and enhanced focal adhesion turnover promote endothelial cell migration. The phosphorylation of Hsp27, a key regulator of actin dynamics, further stimulates actin polymerization, contributing to endothelial cell migration. This signaling cascade exemplifies the role of angiogenic factors in orchestrating cellular processes involved in endothelial cell migration. (KDR: Kinase insert domain receptor; NCK: non-catalytic tyrosine kinase adaptor protein; HSP27: Heat shock protein 27).

VEGF is a potent mediator of vasculogenesis and angiogenesis, partly due to its ability to initiate cell migratory processes. In VEGF-stimulated endothelial cells, ROCK phosphorylation of LIMK leads to actin polymerization and stress fiber rearrangement (Gong et al., 2004). Notably, the induction of cell migration by VEGF involves actin reorganization, including intracellular localized actin polymerization and depolymerization processes. During angiogenesis, VEGF functions as a strong cell chemoattractant for mediating directional endothelial cell migration (Yoshida et al., 1996; Ferrara et al., 2003). Various cellular processes are required for such migration to occur. These include actin polymerization (Rousseau et al., 2000), focal adhesion turnover (Abedi and Zachary, 1997), and changes in cell binding interactions to the ECM that are enhanced by the high-affinity binding of VEGF to its cell surface receptor, kinase insert domain receptor (KDR) (Eliceiri et al., 2002) (Figure 4). In addition, the binding partner of PAK, the non-catalytic tyrosine kinase adaptor protein (NCK), has been shown to play critical roles in mediating focal adhesion turnover to promote cell migration (Stoletov et al., 2001; Gong et al., 2004). The PAK/NCK complex is recruited to the cell surface, where it activates PAK-kinase and promotes PAK redistribution to focal adhesions. Activation of PAK then inhibits actin depolymerization by activating LIMK (Edwards et al., 1999). Indeed, a report by Gong et al. further shows that NCK participates in VEGF-induced regulation of actin dynamics by activating LIMK and promoting cofilin phosphorylation (Gong et al., 2004). NCK regulates actin dynamics by two mechanisms: one includes activation of PAK and the resulting inhibition of F-actin depolymerization via LIMK activation, and the second enhances the activity of actin nucleation-promoting factors leading to the nucleation of nascent actin filaments (Gong et al., 2004). Both mechanisms modulate endothelial cell migration during vascular formation.

Another mechanism by which VEGF promotes endothelial cell migration, proliferation, and tube formation includes the activation of LIMK1 by MAPKAPK-2 (Kobayashi et al., 2006). Kobayashi and colleagues showed that VEGF-stimulated endothelial cells activate LIMK through phosphorylation of LIMK1 by MAPKAPK-2 in a signaling pathway composed of p38-MAPKAPK-2-LIMK1 (Kobayashi et al., 2006). Notably, it was observed that LIMK1 was activated downstream of p38 MAPK by phosphorylation at Ser-323. Furthermore, it was demonstrated that Thr508 was dispensable for LIMK1 activation by VEGF, as a T508V LIMK1 mutant could still phosphorylate cofilin. Moreover, the phosphorylation of Ser-323 in LIMK1 was deemed essential for VEGF-induced cell migration and stress fiber formation, as inhibition of MAPKAPK-2 suppressed VEGF-induced LIMK1 activation and prevented endothelial cell migration (Kobayashi et al., 2006). This is consistent with a previous study that showed the p38-MAPKAPK-2 signaling pathway is involved in VEGF-induced actin reorganization and cell migration through phosphorylation of HSP-27 (Rousseau et al., 2000). Unphosphorylated HSP-27 behaves as an F-actin cap-binding protein that prevents actin polymerization. HSP-27 phosphorylation by MAPKAPK-2 modifies its supramolecular structure. It enables its release from the capped filaments, generating new polymerization sites and allowing the addition of new actin monomers needed to increase actin polymerization (Rousseau et al., 2000). A similar study showed that annexin 1 regulates endothelial cell migration in response to VEGF via p38-MAPKAPK-2 (Cote et al., 2010). Annexin 1 is a protein whose phosphorylation is enhanced by VEGF and impaired by p38 inhibition. It was shown that LIMK1 phosphorylates annexin 1 downstream of the p38-MAPKAPK-2 pathway in response to VEGF. Therefore, the phosphorylation of annexin 1 is considered a vital process required for sustained cell migration and angiogenesis (Martin et al., 2008). This indicates that LIMK regulates endothelial cell migration through the phosphorylation of cofilin and via the phosphorylation of annexin 1 to promote cell movement and vascular formation.

### 3.6 LIMKs and endothelial permeability

The vascular endothelium plays a vital role in regulating tissue fluid balance by forming a semipermeable barrier that allows for the selective exchange of various solutes between the circulation and the interstitial space. The primary determinant of vascular permeability is the integrity of interendothelial junctions, which include adherens junctions, gap junctions, and tight junctions [for review, see Komarova et al. (2017)]. Under inflammatory conditions, maintenance of the interendothelial junctions is impaired, leading to increased vascular permeability. Due to the expression variance of several receptors for inflammatory cytokines in different regions of the vascular tree, microvascular permeability is particularly increased in post-capillary venules under inflammatory conditions (Michel and Curry, 1999). The primary mechanism by which inflammatory mediators increase vascular permeability is by forming gaps between endothelial cells (Spindler et al., 2010). During this process, adherens junctions and tight junctions are disrupted, thereby increasing fluid flow across intercellular gaps. Maintaining endothelial barrier properties also requires a finely tuned balance between actin polymerization and depolymerization, as both hyperpolymerization by jasplakinolide and depolymerization by cytochalasin D increase microvascular permeability *in vivo* (Waschke et al., 2005). Accordingly, a group of GTPases, including RhoA, Cdc42, Rac1, and Rap1 regulate cell adhesion partially by reorganizing the junction-associated cortical actin cytoskeleton (Spindler et al., 2010), which inherently implies the intervention of LIMK activity and cofilin during inflammation-induced vascular permeability (Pober and Sessa, 2007; Skaria et al., 2016).

Recently, the mechanism by which LIMK2 contributes to disrupting barrier function and monolayer integrity through Wnt5A signaling in human coronary artery endothelial cells was determined (Skaria et al., 2017b; Skaria et al., 2017a). Wnt5A is a lipid-modified signaling protein present at high concentrations in the sera and bone marrow of patients with septic shock and severe systemic inflammation (Pereira et al., 2008; Angers and Moon, 2009). Wnt5A targets and activates ROCK to phosphorylate LIMK2 and cofilin, thereby promoting the formation of actin stress fibers. This leads to the disruption of VE-cadherin and β-catenin at adherens junctions, forming interendothelial gaps and increasing the permeability of the endothelial monolayer (Skaria et al., 2017a). Wnt5A acts through the Ryk receptor to affect endothelial barrier function, as suppressing Ryk expression inhibits Wnt5A-induced hyperpermeability (Skaria et al., 2017b). Indeed, it was shown that Ryk-specific Wnt inhibitory factor 1 (WIF1) (Green et al., 2014) inhibited Wnt5A activity and reduced the development of F-actin stress fibers caused by Wnt5A (Skaria et al., 2016; Skaria et al., 2017b). As the cortical actin cytoskeleton spans the entire circumference of endothelial cells and comprises F-actin bundles that are associated with tight junctions and adherens junctions, it is potentially possible to improve endothelial barrier properties by enhancing junctional protein stability through strengthening of cortical actin (Spindler et al., 2010). There is a preponderance of evidence that supports this idea and shows that multiple GTPases use this method to control permeability, most notably Rac1 and Cdc42 (Adamson et al., 2002; Kouklis et al., 2004; Mehta and Malik, 2006; Baumer et al., 2008). Overall, intervening in this mechanism via modulation of LIMK activity may represent a potentially promising approach for treating vascular leakage resulting from inflammation.

## 4 LIMKs roles in the heart

The LIMKs are important participants in cardiomyocyte embryonic development, cardiomyocyte maintenance, and cardiac performance (Li et al., 2012). Furthermore, LIMK activity has been associated with the appropriate formation of cardiac organoids using human pluripotent stem cells in culture (Noh et al., 2023). The role of LIMK in cardiac organoid formation was determined using the potent LIMK inhibitor, LIMKi3, and appeared to involve the appropriate maturation and stabilization of both cardiomyocytes and blood vessels. Indeed, it has been well documented that RhoA-ROCK signaling provides cardioprotective effects. This appears to occur via the activity of LIMK on cofilin in cardiomyocytes (Kilian et al., 2021) in addition to the upstream stimulation of ECM-integrin cascades. As such, the LIMKs have been implicated in both physiological and pathological cardiac phenomena, providing an ample scenario for the roles that LIMKs play in CVD and the potential novel therapeutic use of LIMK inhibition to ameliorate CVD (Table 1).

**TABLE 1:** Representative studies on the role of LIMK in cardiovascular diseases.

CVD	Cells involved	LIMK isoform	References
Diabetes-associated vascular endothelial stiffening (Discussed in section 3.1)	Human umbilical vein endothelial cells	Unknown	Power et al. (2023), Power et al. (2024b)
Inward remodeling (Discussed in section 3.1)	VSMCs	Unknown	Foote et al. (2016)
Hypertension (Discussed in section 3.1)	VSMCs	Unknown	Morales-Quinones et al. (2020)
Intracranial aneurysms (Discussed in section 3.2)	VSMCs	LIMK1	Akagawa et al. (2006)
Vascular cell migration (Discussed in section 3.3 and 3.5)	Human umbilical vein endothelial cells and human aortic smooth muscle cells	LIMK1	Kobayashi et al. (2006), San Martin et al. (2008)
Coronary artery leakage (Discussed in section 3.6)	Human coronary artery endothelial cells	LIMK2	Skaria et al., (2017b), Skaria et al. (2017a)
Atrial fibrillation (Discussed in section 4)	Cardiac fibroblasts	LIMK1	Chen et al. (2019)
Ocular hypertension (Discussed in section 6)	Unknown	LIMK2	Harrison et al. (2009), Harrison et al. (2015)
Thrombsis (Discussed in section 5)	Platelets	LIMK1	Pandey et al. (2006), Antonipillai et al. (2019)

LIMK activity has been particularly implicated in the development of atrial fibrillation and valvular heart disease. Atrial fibrillation is an irregular heart rhythm that begins at the upstream chambers of the heart (atria) and often causes an abnormally fast heart rate. Patients who suffer from recurrent atrial fibrillation are at an increased risk for valvular heart disease. The pathophysiology of atrial fibrillation is characterized by a gradual development of atrial fibrosis and abnormal electrical conduction of ventricular impulses. One key player in the development of atrial fibrillation is the profibrotic molecule, transforming growth factor-β (TGF-β). LIMK1 interacts with TGF-β to modulate actin cytoskeletal dynamics and microtubule stability. In addition, TGFβ1 promotes actin polymerization through the ROCK1-LIMK-cofilin phosphorylation pathway, which can be abolished via ROCK1 inhibition (Vardouli et al., 2005). It has also been shown that TGF-β promotes the differentiation of cardiac fibroblasts into myofibroblasts. Thus, activation of LIMK by TGF-β may be a mechanism by which LIMK promotes cardiac fibrosis. Considering the role of LIMK1 in tissue remodeling, Chen et al. collected clinical data and biopsies of the right atrial appendages from patients with valvular heart diseases to assess the role of LIMK1 in the pathophysiology of atrial fibrillation (Chen et al., 2019). Data from that study showed that LIMK1 expression was greater in patients with atrial fibrillation than in those with sinus rhythm. It was further discovered that LIMK1 downregulation prevents collagen I and collagen II mRNA levels from increasing due to TGF-β interaction with LIMK1. This suggests that increased LIMK1 expression may promote fibrosis and ECM buildup, which could contribute to atrial fibrosis in atrial fibrillation. In addition, TGF-β promotes LIMK1 expression in primary cardiac fibroblasts, and LIMK1 downregulation has been used to prevent TGF-β driven cardiac fibroblast differentiation into myofibroblasts (Chen et al., 2019). Similar results were found in a prior work that demonstrated the downregulation of LIMK1 inhibited fibronectin expression and delayed cell migration in corneal fibroblasts (Gorovoy et al., 2008). LIMK interacts with TGF-β to promote cardiac fibrosis by increasing the deposition of ECM by cardiac fibroblasts and appears to regulate endothelial to mesenchymal transition of atrial endothelium to promote atrial fibrosis further (Lai et al., 2022). Therefore, LIMK inhibitors may represent a promising therapeutic strategy for the amelioration of atrial fibrosis, fibrillation, and associated valvular heart disease.

## 5 LIMKs and microtubules

Microtubules are filamentous polymers comprised of α- and β-tubulin heterodimers that participate in several cellular processes such as mitosis, migration, organelle trafficking, and protein transport (Gundersen and Cook, 1999; Waterman-Storer and Salmon, 1999). These dynamic polymers are regulated by a balance between microtubule-stabilizing and microtubule-destabilizing molecules (Andersen, 2000; Niethammer et al., 2007). In turn, microtubule dynamics can modulate intracellular signal transduction (Gundersen and Cook, 1999). For example, microtubule disruption enhances the formation of F-actin stress fibers and promotes cell contractility (Danowski, 1989). Indeed, it has been found that microtubule disruption by agents such as vinblastine and nocodazole promotes focal adhesion formation and rapid F-actin assembly. In contrast, microtubule stabilization with taxol prevents these effects (Bershadsky et al., 1996). It is also well documented that the Rho GTPases are necessary for microtubule-mediated regulation of the actin cytoskeleton (Wittmann and Waterman-Storer, 2001). However, little is known about the role of LIMK activity in microtubule stability. Some insights into the role of the LIMKs on the interaction between the formation and stability of the actin and microtubular cytoskeleton come from studies on the effects of thrombin in cells. Treatment of cells with thrombin promotes microtubule destabilization, leading to the formation of F-actin stress fibers. Thrombin is known to activate RhoA and ROCK (Essler et al., 1998). In contrast, inhibition of ROCK with Y27632 abolishes the effect of thrombin-induced F-actin stress fiber formation and prevents thrombin-induced destabilization of microtubules (Birukova et al., 2004a). This suggests that thrombin acts through the RhoA-ROCK pathway to promote microtubule destabilization. As mentioned, the LIMKs are downstream targets of ROCK. This implicates them in microtubule destabilization. In support of this thesis, thrombin induces both LIMK1 phosphorylation and cofilin phosphorylation in a time-dependent manner in human pulmonary artery endothelial cells (Leonard et al., 2013). In addition, it has been recently reported in a mouse neuroblast cell line that the destabilization of microtubules with nocodazole results in increased phosphorylation of LIMK1 (Roy et al., 2024). It has also been demonstrated in osteoclasts that nocodazole treatment increases cofilin phosphorylation (Zalli et al., 2016). This suggests a positive feedback loop may exist in which LIMK activation induces microtubule destabilization, and microtubule disruption increases LIMK activation (Gorovoy et al., 2005; Roy et al., 2024). Whether this feedback loop is part of cell biological processes is unknown and, if so, how it is controlled requires further investigation.

As further evidence of the link that microtubule regulation has with actin cytoskeleton reorganization, Gorovoy et al. showed that LIMK1 plays a major role in microtubule destabilization, favoring actin polymerization in human endothelial cells (Gorovoy et al., 2005). They determined that endogenous LIMK1 colocalizes with microtubules to form a complex with tubulin via the LIMK1 PDZ domain. The disruption of microtubules induced by nocodazole, or thrombin, reduced the colocalization of LIMK1 with microtubules but enhanced the interaction of LIMK1 with F-actin. This is consistent with a previous study demonstrating that LIMK inhibition with the compound Pyr1 stabilizes microtubules and abrogates neoplastic cell growth (Prudent et al., 2012). In a similar fashion, siRNA-mediated downregulation of endogenous LIMK1 has been shown to prevent thrombin-induced microtubule destabilization and inhibit thrombin-induced actin polymerization (Gorovoy et al., 2005). Interestingly, the interaction of LIMK1 with tubulin is drastically reduced when ROCK2 is expressed in endothelial cells but increases the interaction between LIMK1 and actin. Consequently, the knockdown of LIMK1 suppresses thrombin-induced microtubule destabilization and F-actin polymerization. This indicates that LIMK1 activity is necessary for orchestrating thrombin-induced actin polymerization and microtubule destabilization. Indeed, the crosstalk between the actin and microtubule cytoskeleton is needed for optimal cellular functions such as migration, cytokinesis, polarity, and locomotion (Rodriguez et al., 2003). Therefore, the findings summarized above provide insight into how LIMK crosstalk with ROCK mediates microtubule disassembly and promotes the formation of F-actin stress fibers.

## 6 LIMK inhibitors as therapeutic agents

As already established, the LIMKs contribute to a broad spectrum of cellular activities, including cell motility, proliferation, differentiation, gene expression, apoptosis, morphogenesis, etc., via the regulation of cytoskeletal dynamics (Scott and Olson, 2007; Prunier et al., 2017). Correspondingly, LIMK activity may be associated with a wide variety of pathologies, including hypertension (Morales-Quinones et al., 2020), aneurysm (Akagawa et al., 2006), erectile dysfunction (Park et al., 2021), glaucoma (Harrison et al., 2015), cancer resistance to microtubule-targeting chemotherapy (Acevedo et al., 2007; Mardilovich et al., 2015), pain (Yang et al., 2017), neuronal diseases (Ben Zablah et al., 2021), neurofibromatosis (Petrilli et al., 2014), etc. At the same time, its inhibition has emerged as a prospective therapeutic target for some of these diverse diseases. Several LIMK inhibitors have been developed for the treatment of conditions such as breast cancer (BMS3, LX7101, Pyr1) (Harrison et al., 2015; Prunier et al., 2016; Malvi et al., 2020), pancreatic cancer (T56-LIMKi) (Rak et al., 2014), Alzheimer’s disease (SR7826) (Henderson et al., 2019), Leukemia (Pyr1) (Prudent et al., 2012), Glaucoma and ocular hypertension (LX7101) (Harrison et al., 2009; Harrison et al., 2015). Although LIMK activity modulation appears to be a potential target for ameliorating the development and progression of cardiovascular disorders, no direct LIMK inhibitors have been approved for the treatment of CVD. Since the first report on the development of a selective inhibitor of LIMK activity by Bristol-Myers-Squibb in 2006, only one such molecule has reached clinical trials for treating glaucoma (Ross-Macdonald et al., 2008). The highly conserved chemical features of kinase ATP binding sites often make finding or developing selective LIMK inhibitors challenging (Simard and Rauh, 2014). Therefore, substances that interact with the ATP binding pocket in the LIMKs, such as type I and type II inhibitors, frequently have the same inhibitory activity across many kinases with little to no selectivity. Other novel compounds have been developed using a wide range of chemical scaffolds for LIMK inhibition, with excellent results (Manetti, 2012; Berabez et al., 2022; Bukhari et al., 2022). However, most of the LIMK inhibitors available today are type 1 kinase inhibitors that bind to the active conformation of the LIMKs in the ATP pocket (Manetti, 2012; Bukhari et al., 2022).

Owing to the role of the LIMKs in regulating cytoskeletal dynamics and their position as downstream effectors of numerous signaling cascades, we consider LIMK inhibition to be a promising approach for the treatment of cardiovascular disorders. Notably, it has been shown that LIMK inhibition destabilizes the intracellular cytoskeleton of platelets, thereby inhibiting platelet aggregation and adhesion to fibrinogen hence, promoting thrombolysis (Estevez et al., 2013; Antonipillai et al., 2019). Recently, Morales-Quinones et al. used the cell-permeable and potent LIMK inhibitor, LIMKi3, as a tool to stop the progression of arterial remodeling (Morales-Quinones et al., 2020). LIMKi3 effectively suppresses cellular LIMK-associated cofilin phosphorylation without affecting tubulin polymerization or inducing cytotoxicity. LIMK inhibition thus activates cofilin and promotes F-actin stress fiber depolymerization. In agreement with this, Morales-Quinones et al. found that LIMK inhibition reduced the stiffness caused by prolonged exposure to the vasoconstrictor agonists norepinephrine and angiotensin II in human VSMCs in culture and isolated human arteries under pressure myography. LIMKi3 also ameliorated arterial inward remodeling in mice with angiotensin II-induced hypertension (Morales-Quinones et al., 2020). Similarly, Foote et al. demonstrated that LIMK inhibition prevented the inward remodeling associated with the exposure of isolated arteries to the vasoconstrictor agonist serotonin combined with the nitric oxide synthase competitive inhibitor L-NAME (Foote et al., 2016). These studies provide strong evidence for considering LIMK inhibition as a potential therapeutic avenue to ameliorate aberrant arterial remodeling and its associated cardiovascular diseases.

More recent studies have been conducted on the use of LIMK inhibition for the treatment of multiple diseases. Several LIMK inhibitors have been synthesized and used to reduce intraocular pressure (Harrison et al., 2009; Boland et al., 2015; Yin et al., 2015). ROCK inhibitors can be used for that purpose, but they are associated with several side effects (Tanihara et al., 2008). This is overcome using the pyrrolopyrimidine class of LIMK 2 inhibitors, effectively lowering ocular pressure and associated glaucoma with reduced side effects (Harrison et al., 2009; Harrison et al., 2015). Berabez and colleagues recently reviewed several LIMK inhibitors that have progressed to the preclinical trial stage. However, none of them are specifically intended for the treatment of cardiovascular disorders (Berabez et al., 2022). New LIMK inhibitors have been developed, and their characteristics, including their selectivity, cell biological activity, and efficiency, have been described (Ariawan et al., 2021; Rybin et al., 2022; Champire et al., 2024). This furthers the notion that LIMK inhibition has promising therapeutic potential for multiple disease conditions. Some of the main concerns reported as preventing the advancement of LIMK into clinical trials are addressed with the new inhibitors (Manetti, 2018; Berabez et al., 2022; Champire et al., 2024). These improvements include a better selectivity of the inhibitors presenting little or no effects on kinases other than the LIMKs and even prefer one of the LIMKs, which would allow them to target cellular processes specific to one of the LIMK isoforms (Berabez et al., 2022; Champire et al., 2024). The capacity or incapacity of the LIMK inhibitors to cross the blood-brain barrier has also been a concern. In this regard, the inability of a compound to cross the blood-brain barrier may be an advantage for using LIMK inhibition to treat vascular diseases such as endothelial dysfunction, as the inability of compounds to cross the blood-brain barrier would limit neurological effects while maintaining endothelial targeting. Although all LIMK and cofilin isoforms appear to be expressed in smooth muscle, endothelium, and cardiomyocytes (Boengler et al., 2003; Bongalon et al., 2004; Pipp et al., 2004; Gorovoy et al., 2005; Goyal et al., 2005; Acevedo et al., 2006; Dai et al., 2006; Goyal et al., 2006; Kobayashi et al., 2006; Fazal et al., 2009; Cote et al., 2010; Lindstrom et al., 2011; Subramanian et al., 2015; Wang et al., 2016; Chatzifrangkeskou et al., 2018; Yu et al., 2021; Le Dour et al., 2022; Du et al., 2024), the level of expression of each enzyme isoform in different regions of the vascular tree, the heart, or specific cell types within those regions has not been thoroughly characterized. Such information would help choose the appropriate LIMK inhibitor to target a particular cellular type and pathophysiological process. This should be followed by testing the selected inhibitors in relevant preclinical animal models of cardiovascular diseases that involve actin polymerization derangements. Overall, research on the effects of LIMK inhibition indicates that LIMK inhibitors are still waiting to be harnessed in clinical settings and can potentially become effective treatments for cardiovascular disorders.

## 7 Conclusion

The LIMKs play preponderant roles in multiple cellular mechanisms. Due to the role that LIMK activity plays in cytoskeletal dynamics, it is considered particularly prominent in phenomena associated with cell migration, contraction, and stiffening. In the cardiovascular system, these phenomena are related to pathophysiological processes such as vascular remodeling, stiffening, permeability, angiogenesis, atrial stiffening, and fibrillation. Several LIMK inhibitors have been developed over the years, but these have been primarily tested as therapeutic agents for reducing intraocular pressure and cancer. Although the inhibition of LIMK activity should be considered a potential avenue for treating several cardiovascular diseases, their efficacy and tolerance as therapeutic agents for this purpose remains to be determined.
